# Impact of serum carotenoids on cardiovascular mortality risk in middle-aged and elderly adults with metabolic syndrome

**DOI:** 10.3389/fnut.2024.1465972

**Published:** 2024-11-13

**Authors:** Jing Han, Ruiyun Wang, Lijuan Bai, Yun Liu, Man Liao, Liting Zhang, Lihua Liu, Benling Qi

**Affiliations:** Department of Geriatrics, Union Hospital, Tongji Medical College, Huazhong University of Science and Technology, Wuhan, Hubei Province, China

**Keywords:** cardiovascular mortality, carotenoids, antioxidant, metabolic syndrome, NHANES

## Abstract

**Background:**

Metabolic syndrome (MetS), characterized by abdominal adiposity, hypertension, hyperglycemia, and dyslipidemia, is associated with dysregulated immune function, elevated oxidative stress, and chronic low-grade inflammation. Aging exacerbates insulin resistance and the prevalence of MetS. Dietary antioxidants, such as carotenoids, may play a role in preventing cardiovascular disease (CVD) mortality, but evidence remains mixed, particularly among middle-aged and elderly individuals with MetS.

**Methods:**

We analyzed data from 6,601 participants aged 40 years and above with MetS from the National Health and Nutrition Examination Survey (NHANES) III (1988–1994) and NHANES 2001–2006 cycles. Serum concentrations of *α*-carotene, *β*-carotene, lycopene, β-cryptoxanthin, and combined lutein/zeaxanthin were quantified. Participants were followed for a median of 16.8 years. Cox proportional-hazards models were used to assess the association between serum carotenoid concentrations and CVD mortality risk, with adjustment for potential confounders.

**Results:**

During the follow-up period, 1,237 CVD deaths were identified. Analysis revealed an inverse dose–response relationship between serum lycopene levels and cardiovascular mortality risk. Compared to the lowest quartile, the multivariable-adjusted hazard ratios (95% confidence intervals) for ascending quartiles of serum lycopene were 0.84 (0.71, 1.00), 0.87 (0.74, 1.03), and 0.77 (0.61, 0.97), with a significant trend (*p* = 0.039). No significant associations were observed for other carotenoids.

**Conclusion:**

In this prospective cohort study of 40-year-old and older individuals with MetS, we observed an inverse association between serum lycopene levels and CVD mortality risk.

## Introduction

Metabolic syndrome (MetS) is a significant public health concern characterized by a cluster of interrelated conditions, including abdominal obesity, hypertension, hyperglycemia, and dyslipidemia (1). These factors collectively increase the risk of developing cardiovascular diseases (CVD) and type 2 diabetes mellitus(T2DM) (2, 3). Notably, the prevalence of MetS continues to rise especially among countries with fast-aging populations (4). Epidemiological data indicate that approximately one-third of the adult population in the United States is affected by MetS, with prevalence rising to nearly half among individuals aged 60 years and above (5). This demographic is vulnerable due to the synergistic effects of aging, which exacerbate metabolic dysregulation and contribute to chronic low-grade inflammation, thereby increasing the risk of cardiovascular events (6–11). Epidemiological studies indicate that antioxidant nutrients may contribute to the prevention of CVD mortality (12).

Carotenoids are common natural antioxidants, among which lycopene, *β*-carotene, β-cryptoxanthin, *α*-carotene, lutein, and zeaxanthin account for over 95% of the total carotenoid concentration in human blood (13). Recent observational studies have indicated that dietary carotenoid intake and serum carotenoid levels are inversely associated with the prevalence and progression of MetS and its components (14–16). However, the results across these studies have not been entirely consistent (17). The impact of carotenoids, particularly *β*-carotene supplementation, on cardiovascular disease (CVD) mortality remains equally ambiguous (13, 18–20). While some observational studies and interventional trials have reported mixed outcomes across diverse populations, higher serum carotenoid concentrations are generally linked to reduced risks of all-cause and cardiovascular mortality in the general population, as well as among adults with hypertension and metabolic dysfunction-related fatty liver disease (18, 20, 21). Conversely, *β*-carotene has been associated with an increased risk of cardiovascular mortality in individuals with T2DM and smokers (13, 22). This is particularly concerning given that MetS closely relates to hypertension and T2DM. Among middle-aged and elderly individuals affected by MetS, evidence elucidating the potential cardiovascular effects of carotenoids is still scarce (23, 24). Moreover, it remains unclear whether factors such as sex, age, renal function and smoking status might modify the associations between carotenoid levels and cardiovascular outcomes.

Thus, we conducted a prospective investigation to investigate the relationship between serum carotenoid concentrations and CVD mortality among middle-aged and older adults with MetS. This study utilized data from a nationally representative sample of the U.S. population, with a median follow-up duration of 16.8 years.

## Methods

### Study participants

The NHANES, a comprehensive epidemiological program, has been assessing the health and dietary patterns of the U.S. population since the early 1960s. Using a multistage, stratified, clustered sampling design, NHANES recruits a nationally representative sample, examining approximately 5,000 individuals annually. Our study focused on participants from NHANES III and Continuous NHANES (2001–2006), as these cycles included serum carotenoid measurements. We selected individuals aged 40 years and older with MetS who had complete data on five serum carotenoids, resulting in a final cohort of 8,684 subjects. We excluded 76 individuals with no follow-up information, 818 participants with cancer, 1,098 participants with CVD, 1 participant self-reported as pregnant, 90 individuals with extreme energy intake(defined as <500 kcal/d for both sexes, >5,000 kcal/d for women, or > 8,000 kcal/d for men) (25). Finally, 6,601 participants were analyzed in the present study (Supplementary Figure S1).

### Ascertainment of MetS

We identified the MetS according to the 2005 National Cholesterol Education Program Adult Treatment Panel III criteria (26). Participants were classified as having MetS if they had at least three of the following five criteria: 1. Central obesity: waist circumference ≥ 88 cm for women or ≥ 102 cm for men, or body mass index (BMI) >30 kg/m^2^; 2. Elevated fasting plasma glucose: ≥100 mg/dL or use of glucose-lowering agents; 3. Hypertriglyceridemia: triglycerides ≥150 mg/dL or receiving treatment; 4. Low high-density lipoprotein cholesterol (HDL-C): <50 mg/dL in women or < 40 mg/dL in men, or receiving treatment; 5. Hypertension: systolic blood pressure ≥ 130 mmHg or diastolic blood pressure ≥ 85 mmHg, or antihypertensive medication use.

### Ascertainment of carotenoids

Serum concentrations of five carotenoids (*α*-carotene, *β*-carotene, lutein/zeaxanthin, lycopene, and β-cryptoxanthin) were assayed using high-performance liquid chromatography (HPLC) in NHANES III and NHANES cycles 2001–2002 and 2005–2006. In NHANES 2003–2004, a comparable HPLC method was employed, and the data were adjusted using regression analysis to obtain equivalent carotenoid measurements based on HPLC. Cumulative serum carotenoid concentrations were obtained by combining the individual measurements of five carotenoids. Detailed measurements for serum carotenoids are accessible in the NHANES Laboratory Methods documentation.^1^

### Ascertainment of mortality

Participants’ mortality status was determined by linking their records to the National Death Index using unique identifiers, up to December 31, 2019. Causes of death were categorized based on the International Classification of Diseases, 10th Revision (ICD-10). The primary endpoint was CVD mortality, defined as deaths attributed to codes I00-I09, I11, I13, I20-I51, and I60-I69, which encompass a range of cardiovascular and cerebrovascular conditions.

### Ascertainment of covariates

Standardized questionnaires were employed to gather detailed information on individual demographics, including age, sex, race/ethnicity, and education level. Socioeconomic factors such as poverty status, as well as lifestyle behaviors including smoking status, alcohol consumption, physical activity, and dietary intake, were also recorded. Additionally, data on dietary supplement use (specifically vitamins and minerals), BMI, and the use of medications for diabetes, hypertension, and hyperlipidemia were collected. Smoking status was categorized as follows: current smokers were those who reported a lifetime consumption of at least 100 cigarettes and continued to smoke; former smokers had the same lifetime consumption but had ceased smoking; and non-smokers had consumed fewer than 100 cigarettes in their lifetime. Alcohol consumption was classified as nondrinker, low-to-moderate (<1 drink/d for women, <2 drinks/d for men), or heavy (≥1 drink/d for women, ≥2 drinks/d for men). Physical activity levels were determined by self-reported leisure-time exercise, with participants categorized as inactive (no reported activity), insufficiently active (engaging in moderate-intensity activities 1–5 times weekly, or vigorous activities 1–3 times weekly), or active (exceeding the insufficiently active criteria) (27). Renal function was assessed using the estimated glomerular filtration rate (eGFR), calculated with the CKD-EPI formula (28). Further details of these variables can be found.^2^

### Statistical analysis

According to analytical guidelines, all analyses incorporated primary sampling units, weighting, and strata to provide reliable national estimates. Baseline characteristics were summarized according to quartiles of serum lycopene, with mean (SEs) for normally distributed continuous variables, medians (interquartile ranges) for non-normally distributed continuous variables, and numbers (percentages) for categorical variables. We used one-way ANOVA test, Mann–Whitney U test, and chi-squared test to compare groups for normally distributed continuous, non-normally distributed continuous, and categorical variables, respectively. To assess the association between serum carotenoid levels and cardiovascular mortality risk, we employed multivariable Cox proportional hazards regression models. Concentrations of the five carotenoids were log2-transformed and subsequently categorized into quartiles. We fitted two statistical models: Model 1 adjusted for age, sex, race/ethnicity, education level, alcohol consumption status, smoking status, family poverty income ratio, physical activity, supplement use, total energy intakes (in quartiles), eGFR, and BMI. Model 2 additionally adjusted for systolic blood pressure (continuous), non-high-density lipoprotein cholesterol (continuous), fasting glucose (continuous), Mets components (3–5), insulin use, diabetic pills use, antihypertensive medication use, and antihyperlipidemic drug use. We verified the proportional hazards assumption using Schoenfeld residuals. Furthermore, Kaplan–Meier (KM) curves were employed to illustrate the varying survival probabilities among participants based on different serum carotenoid levels. In addition, restricted cubic spline (RCS) analysis, with knots positioned at the 10th, 50th, and 90th percentiles of serum carotenoid distribution, was conducted to assess potential non-linear relationships. The presence of non-linearity was evaluated using the ANOVA.

To examine potential effect modification, we performed stratified analyses using likelihood ratio tests. Stratification variables included sex (female or male), age (≤60 or > 60 years), smoking status (current or never/past), physical activity status (inactive or active/insufficient), eGFR (≤60 or > 60 mL/min/1.73m^2^), and the number of MetS components (3, 4, or 5).

To assess the robustness of the findings, we conducted three sensitivity analyses. First, to reduce the likelihood of reverse causality, we excluded individuals who died within the initial 2 years of follow-up, consistent with previous studies (13, 18). Subsequently, we expanded our models to account for additional dietary factors, incorporating quartiles of total protein, fat, cholesterol, fiber, folate, and vitamins A, E, B12, and C intake. In our final analysis, we further adjusted for serum nutrient biomarkers, including vitamins A, C, D, and E (all in quartiles). Missing covariate data were multiply imputed using the “mice” package in R. Analyses were performed on both the full imputed dataset and a subset excluding imputed values to assess imputation quality. The results presented are based on the full imputed dataset. Results excluding imputed values are not shown but were consistent with the main findings. All data management and statistical analyses were conducted using R statistical software (version 4.3.1). Statistical significance was defined as a two-tailed *p* value below 0.05.

## Results

From the 8,896 MetS participants in the NHANES III and NHANES 2001–2006 cycles, 6,601 individuals met the study’s inclusion criteria. Among these, females accounted for 53.38% (weighted percentage), with a mean age of 56 years. The median (interquartile range) serum concentrations were 0.06 (0.03, 0.10) μmol/L for *α*-carotene, 0.25 (0.15, 0.42) μmol/L for *β*-carotene, 0.13 (0.08, 0.20) μmol/L for β-cryptoxanthin, 0.37 (0.24, 0.52) μmol/L for lycopene, and 0.29 (0.20, 0.42) μmol/L for lutein/zeaxanthin. Table 1 presents a comparative analysis of participant characteristics stratified by quartiles of serum lycopene. Participants exhibiting higher serum lycopene levels were generally younger and more likely to be male, non-Hispanic whites, and low-to-moderate alcohol consumers. These individuals also exhibited favorable socioeconomic indicators, including a higher family income-to-poverty ratio and greater educational attainment, alongside increased leisure-time physical activity and higher energy intake levels. Furthermore, these individuals demonstrated a lower frequency of insulin use and lower systolic blood pressure (SBP). In contrast, they exhibited higher diastolic blood pressure (DBP), elevated serum triglycerides, non-high-density lipoprotein cholesterol (non-HDL-C), and total cholesterol levels, as well as increased use of antihyperlipidemic medications. Importantly, participants with elevated serum lycopene concentrations had fewer components of MetS, lower fasting plasma glucose (FPG), and higher eGFR. The baseline distributions and concentrations of the five carotenoids are detailed in Supplementary Table S1. Among these, lycopene exhibited the highest mean concentration at 0.400 μmol/L, followed by *β*-carotene at 0.350 μmol/L, lutein/zeaxanthin at 0.337 μmol/L, β-cryptoxanthin at 0.158 μmol/L, and *α*-carotene at 0.079 μmol/L.

**Table 1:** Baseline characteristics of middle-aged and elderly adults with metabolic syndrome in NHANES III and NHANES 2001–2006.

			Serum lycopene (μmol/L)			
Characteristic	Overall	Q1	Q2	Q3	Q4	*p* value
		<0.242	0.242–0.373	0.373–0.522	>0.522	
Patients, n	6,601	2,075	1,820	1,438	1,268	
Age, years	56 (47.0, 67.0)	62 (51.0, 72.0)	57 (49.0, 67.0)	54 (46.1, 64.0)	53 (45.0, 62.0)	<0.001
Female, n (%)	3,647 (53.4%)	1,192 (58.9%)	1,039 (55.8%)	785 (53.8%)	631 (44.6%)	<0.001
Non-Hispanic White, n (%)	3,249 (77.8%)	937 (72.6%)	863 (77.9%)	742 (79.6%)	707 (81.1%)	<0.001
Smoking status, n (%)						0.429
Current smoker	1,263 (20.0%)	429 (22.0%)	335 (18.9%)	274 (20.6%)	225 (18.4%)	
Former smoker	2,132 (32.9%)	673 (32.1%)	580 (34.2%)	446 (31.3%)	433 (34.0%)	
Never smoker	3,206 (47.1%)	973 (45.9%)	905 (47.0%)	718 (48.0%)	610 (47.6%)	
Drinking status, n (%)						<0.001
Heavy drinker	181 (2.8%)	61 (3.4%)	54 (3.3%)	33 (2.2%)	33 (2.3%)	
Low-to-moderate drinkers	2,905 (50.2%)	777 (41.6%)	793 (49.4%)	666 (53.6%)	669 (56.9%)	
Nondrinker	3,515 (47.0%)	1,237 (55.0%)	973 (47.3%)	739 (44.3%)	566 (40.8%)	
Family income-to-poverty ratio, n (%)						<0.001
≤1	1,633 (15.1%)	613 (20.9%)	472 (14.3%)	310 (12.9%)	238 (12.0%)	
1.1–3.0	2,769 (37.0%)	959 (43.3%)	738 (36.5%)	600 (36.9%)	472 (31.2%)	
>3	2,199 (47.9%)	503 (35.8%)	610 (49.2%)	528 (50.2%)	558 (56.8%)	
Educational attainment, n (%)						<0.001
College or above	1,940 (41.5%)	425 (30.1%)	505 (39.4%)	503 (47.9%)	507 (49.4%)	
High School or equivalent	1,783 (31.2%)	492 (30.1%)	496 (32.5%)	410 (30.7%)	385 (31.7%)	
Less than high school	2,878 (27.3%)	1,158 (39.8%)	819 (28.1%)	525 (21.5%)	376 (18.9%)	
Physical activity, n (%)						<0.001
Active	1,612 (26.7%)	430 (22.5%)	450 (27.6%)	375 (27.9%)	357 (29.1%)	
Insufficiently active	3,608 (48.9%)	1,274 (56.3%)	1,005 (49.3%)	740 (47.5%)	589 (42.0%)	
Inactive	1,381 (24.4%)	371 (21.2%)	365 (23.2%)	323 (24.6%)	322 (28.9%)	
Energy intake, kcal/d	1,845.0 (1,377.0, 2,439.5)	1,645.9 (1,246.5, 2,208.5)	1,786.0 (1,373.0, 2,431.8)	1,902.1 (1,409.8, 2,454.2)	2,091.5 (1,547.0, 2,730.6)	<0.001
Body mass index, kg/m^2^	30.2 (27.1, 33.9)	29.6 (26.4, 33.5)	30.6 (27.2, 34.4)	30.4 (27.2, 34.0)	30.1 (27.4, 33.7)	0.011
eGFR, mL/min per 1.73 m^2^	72.6 (60.0, 87.2)	68 (55.5, 83.6)	72.1 (59.7, 86.2)	76.2 (62.1, 88.7)	76.3 (63.8, 90.5)	<0.001
Serum triglycerides, mg/dL	181 (136.0, 242.1)	172 (129.0, 235.0)	177 (133.0, 234.0)	186 (143.7, 243.0)	191.6 (144.0, 258.7)	<0.001
Serum total cholesterol, mg/dL	216 (188.5, 246.0)	205 (176.0, 234.0)	209 (185.0, 238.9)	220 (194.0, 245.0)	233 (205.0, 264.0)	<0.001
Fasting Glucose, mg/dL	108.3 (97.3, 130.1)	112.2 (99.0, 137.9)	109.5 (97.7, 132.9)	107 (96.4, 126.2)	105.9 (96.2, 125.7)	<0.001
HDL-C, mg/dL	43 (37.0, 51.0)	42 (36.0, 51.0)	42 (37.0, 50.0)	43 (37.0, 52.0)	43 (37.0, 52.0)	0.241
non-HDL-C, mg/dL	171.0 (143.0, 201.0)	159.0 (131.0, 189.0)	163.0 (139.0, 194.0)	173.0 (147.0, 198.6)	188.0 (160.0, 218.0)	<0.001
Systolic blood pressure, mmHg	132 (120.0, 144.7)	134 (121.0, 147.3)	132.7 (121.3, 145.3)	132 (120.7, 143.0)	130.5 (118.0, 142.0)	<0.001
Diastolic blood pressure, mmHg	76 (68.7, 84.0)	74 (67.3, 82.4)	75.3 (68.0, 83.3)	77.3 (68.7, 84.1)	77.3 (70.7, 84.7)	<0.001
Supplement use, n (%)	3,045 (51.6%)	933 (50.5%)	838 (52.4%)	656 (51.4%)	618 (52.3%)	0.890
Diabetic pills use, n (%)	871 (10.9%)	300 (11.9%)	250 (11.3%)	168 (9.7%)	153 (10.6%)	0.414
Insulin use, n (%)	263 (2.1%)	118 (3.6%)	59 (1.4%)	42 (2.0%)	44 (1.5%)	0.001
Antihypertensive medication use, n (%)	2,769 (41.2%)	894 (43.8%)	755 (41.3%)	604 (39.8%)	516 (39.9%)	0.349
Antihyperlipidemic medication use, n (%)	1,598 (27.5%)	450 (25.9%)	422 (25.8%)	359 (26.8%)	367 (31.7%)	0.019
MetS components, n (%)						0.016
3	3,288 (49.7%)	1,001 (46.4%)	876 (48.2%)	731 (49.7%)	680 (54.5%)	
4	2,349 (36.4%)	752 (37.7%)	653 (36.4%)	514 (37.5%)	430 (34.0%)	
5	964 (13.9%)	322 (15.9%)	291 (15.4%)	193 (12.8%)	158 (11.4%)	
α-carotene, μmol/L	0.06 (0.03, 0.10)	0.04 (0.02, 0.07)	0.06 (0.03, 0.09)	0.07 (0.04, 0.11)	0.07 (0.04, 0.12)	<0.001
β-carotene, μmol/L	0.25 (0.15, 0.42)	0.19 (0.11, 0.35)	0.24 (0.15, 0.39)	0.26 (0.17, 0.42)	0.33 (0.20, 0.51)	<0.001
β-cryptoxanthin, μmol/L	0.13 (0.08, 0.20)	0.09 (0.06, 0.16)	0.11 (0.08, 0.18)	0.13 (0.09, 0.20)	0.16 (0.11, 0.24)	<0.001
Lutein/zeaxanthin, μmol/L	0.29 (0.20, 0.42)	0.25 (0.17, 0.37)	0.28 (0.19, 0.38)	0.30 (0.21, 0.41)	0.34 (0.25, 0.48)	<0.001
Total carotenoids, μmol/L	1.19 (0.87, 1.62)	0.80 (0.60, 1.12)	1.05 (0.84, 1.37)	1.27 (1.02, 1.59)	1.65 (1.36, 2.07)	<0.001

Normally distributed continuous variables are described as mean ± SE, and continuous variables without a normal distribution are presented as median (interquartile range). Categorical variables are presented as numbers (percentages). N reflects the study sample while percentages reflect the survey-weighted. eGFR, estimated glomerular filtration rate; HDL-C, high-density lipoprotein cholesterol; non-HDL-C, non-high-density lipoprotein cholesterol.

During a mean follow-up time of 16.8 years, 1,237 subjects with CVD deaths among 6,601 MetS middle-aged and older adults were identified. Table 2 shows the associations between the risk of CVD mortality in MetS middle-aged and older adults and the levels of 5 serum carotenoids, as assessed through 3 multiple Cox regression analyses. After making adjustments for model 1, the serum lycopene concentrations were observed to have substantial correlations with CVD mortality. With further adjustments for model 2, the findings remained stable and statistically significant. The multivariable-adjusted hazard ratios (95% CIs) across quartiles of serum lycopene were 1.00 (reference), 0.84 (0.71, 1.00), 0.87 (0.74, 1.03), and 0.77 (0.61, 0.97; P trend = 0.039). Notably, compared to the reference quartile, the third and fourth quartiles of *β*-carotene exhibited an increased risk of CVD mortality in crude models; however, after additional adjustments, no significant associations were found between CVD mortality risk and serum levels of β-cryptoxanthin or β-carotene. Additionally, a significant association was observed between the second quartile of *α*-carotene (HR, 0.76 [95% CI, 0.61–0.94]) and lutein/zeaxanthin (HR, 0.79 [95% CI, 0.62–0.99]) with CVD mortality when compared to the first quartile after multivariate adjustment; whereas no significant associations were found for the third and fourth quartiles of α-carotene or lutein/zeaxanthin. Similar associations were observed between total serum carotenoid levels and CVD mortality, as detailed in Supplementary Table S2.

**Table 2:** Hazard ratios (95% CIs) of CVD according to quartiles of serum carotenoids concentrations among middle-aged and elderly adults with MetS in NHANES III and NHANES 2001–2006.

	Serum carotenoids (μmol/L)				
	Quartile 1	Quartile 2	Quartile 3	Quartile 4	*P* trend
α-Carotene					
Range	<0.032	0.032–0.058	0.058–0.097	>0.097	
No. deaths/total	263/1551	274/1650	348/1736	352/1664	
Crude	1	0.91 (0.72, 1.14)	1.05 (0.85, 1.29)	1.12 (0.91, 1.37)	0.164
Model 1	1	0.78 (0.63, 0.98)	0.88 (0.69, 1.10)	0.82 (0.65, 1.03)	0.214
Model 2	1	0.76 (0.61, 0.94)	0.84 (0.67, 1.06)	0.81 (0.64, 1.04)	0.217
β-Carotene					
Range	<0.149	0.149–0.252	0.252–0.421	>0.421	
No. deaths/total	235/1551	254/1551	331/1685	417/1814	
Crude	1	1.08 (0.81, 1.43)	1.29 (1.01, 1.65)	1.52 (1.22, 1.88)	<0.001
Model 1	1	0.92 (0.67, 1.25)	0.98 (0.75, 1.30)	0.86 (0.65, 1.14)	0.399
Model 2	1	0.89 (0.65, 1.23)	1.02 (0.79, 1.33)	0.90 (0.69, 1.17)	0.652
β-Cryptoxanthin					
Range	<0.081	0.081–0.127	0.127–0.199	>0.199	
No. deaths/total	248/1338	334/1685	275/1530	380/2048	
Crude	1	0.99 (0.79, 1.24)	0.85 (0.68, 1.08)	0.99 (0.81, 1.20)	0.542
Model 1	1	1.05 (0.83, 1.31)	0.90 (0.70, 1.14)	0.87 (0.70, 1.09)	0.118
Model 2	1	1.02 (0.81, 1.29)	0.87 (0.69, 1.11)	0.85 (0.68, 1.07)	0.070
Lycopene					
Range	<0.242	0.242–0.373	0.373–0.522	>0.522	
No. deaths/total	472/2075	330/1820	251/1438	184/1268	
Crude	1	0.63 (0.52, 0.75)	0.50 (0.41, 0.60)	0.40 (0.32, 0.49)	<0.001
Model 1	1	0.84 (0.72, 1.00)	0.85 (0.72, 1.00)	0.77 (0.62, 0.96)	0.026
Model 2	1	0.84 (0.71, 1.00)	0.87 (0.74, 1.03)	0.77 (0.61, 0.97)	0.039
Lutein/zeaxanthin					
Range	<0.204	0.204–0.290	0.290–0.418	>0.418	
No. deaths/total	190/1269	258/1570	311/1758	478/2004	
Crude	1	1.02 (0.77, 1.35)	0.98 (0.77, 1.24)	1.30 (1.00, 1.70)	0.089
Model 1	1	0.82 (0.64, 1.04)	0.79 (0.62, 1.01)	0.92 (0.72, 1.16)	0.475
Model 2	1	0.79 (0.62, 0.99)	0.77 (0.60, 1.00)	0.85 (0.65, 1.12)	0.299

Model 1 was adjusted for age (continuous), sex (male or female), race (non-Hispanic white or other), education level (less than high school, high school or equivalent, or college or above), smoking status (never smoker, former smoker, or current smoker), alcohol consumption status (nondrinker, low-to-moderate drinker, or heavy drinker), family poverty income ratio (<1.0, 1.0–3.0, or > 3.0), physical activity (inactive group, insufficiently active group, or active group), supplement use (yes or no), total energy intakes (in quartiles), eGFR (continuous), BMI(continuous). Model 2 was adjusted for as model 1 plus SBP (continuous), serum non-HDL-C (continuous), fasting glucose (continuous), Mets components (3–5), insulin use (yes or no), diabetic pills use (yes or no), antihypertensive medication use (yes or no), antihyperlipidemic drug use (yes or no).

Additionally, KM survival analysis showed that higher concentrations of lycopene were significantly associated with improved survival (Log-rank *p* < 0.0001). A similar trend was observed for lutein/zeaxanthin (Log-rank *p* = 0.011). In contrast, lower concentrations of *β*-carotene were linked to significantly higher survival rates (Log-rank *p* < 0.0001). No significant differences in survival were noted for *α*-carotene and β-cryptoxanthin (Log-rank *p* > 0.05; Supplementary Figure S2).

**Figure 1:**
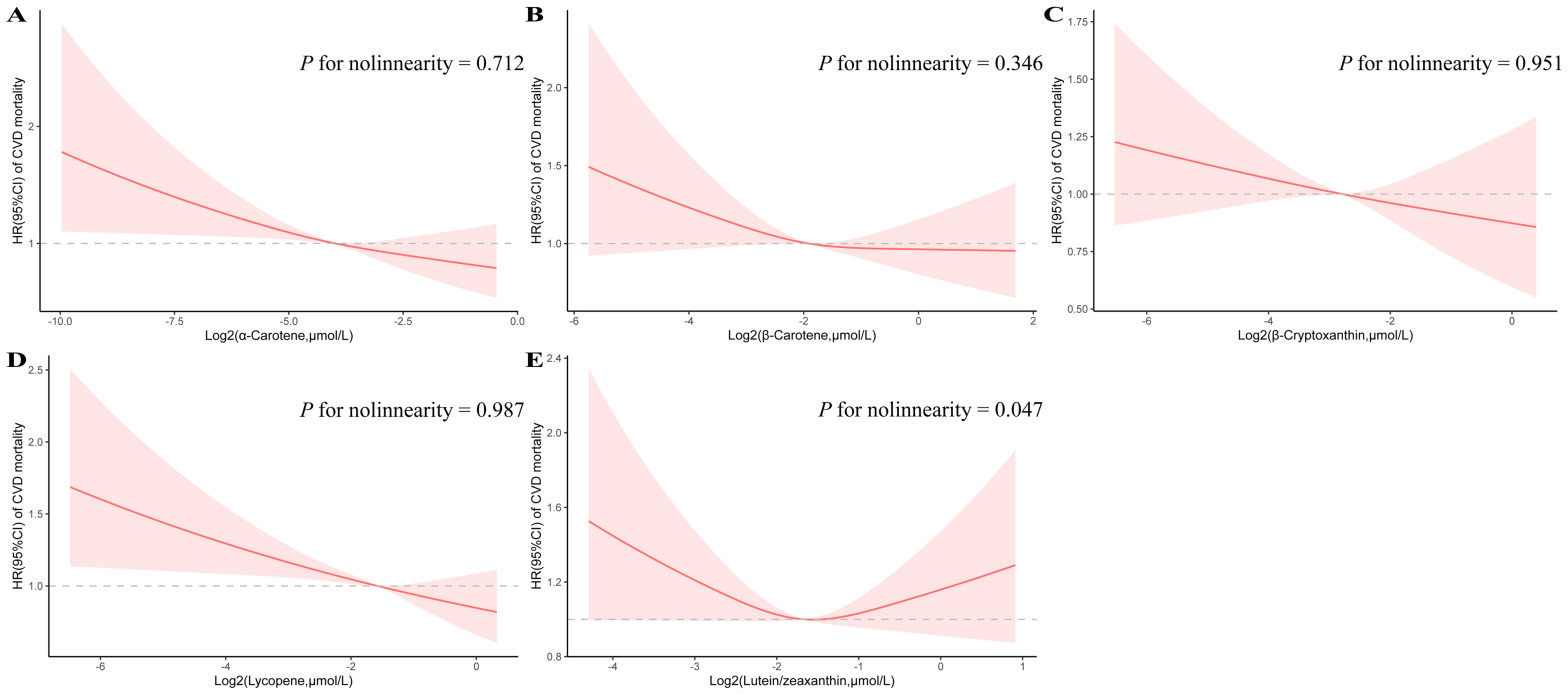
Restricted cubic spline analyses of the association of serum carotenoids (A: *α*-carotene, B: *β*-carotene, C: β-cryptoxanthin, D: lycopene, and E: lutein/zeaxanthin) with CVD mortality. Adjusted for age (continuous), sex (male or female), race (non-Hispanic white or other), education level (less than high school, high school or equivalent, or college or above), smoking status (never smoker, former smoker, or current smoker), alcohol consumption status (nondrinker, low-to-moderate drinker, or heavy drinker), family poverty income ratio (<1.0, 1.0–3.0, or > 3.0), physical activity (inactive group, insufficiently active group, or active group), supplement use (yes or no), total energy intakes (in quartiles), eGFR (continuous), BMI(continuous), SBP (continuous), serum non-HDL-C (continuous), fasting glucose (continuous), Mets components (3–5), insulin use (yes or no), diabetic pills use (yes or no), antihypertensive medication use (yes or no), antihyperlipidemic drug use (yes or no).

RCS plots were performed to assess the potential non-linearity of the association between CVD mortality in MetS subjects and the log2-transformed carotenoids (Figure 1). Of note, a U-shaped relationship between the log2-transformed serum lutein/zeaxanthin levels and CVD mortality could be observed (P for nonlinearity = 0.047). For the other major carotenoids examined, including *α*-carotene, *β*-carotene, β-cryptoxanthin, and lycopene, the associations with CVD mortality exhibited a linear decreasing trend.

The inverse association between serum lycopene levels and CVD mortality risk persisted across stratified analyses by age (≤60 or > 60 years), sex (female or male), smoking status (current or never/past), physical activity status (inactive or active/insufficient), eGFR (≤60 or > 60 mL/min/1.73m^2^), and number of metabolic syndrome components (3, 4, or 5), as delineated in Table 3. No significant interactions were detected after adjusting for multiple comparisons. Additionally, subgroup analyses examining the relationships between serum levels of *α*-carotene, *β*-carotene, β-cryptoxanthin, lutein/zeaxanthin, and total carotenoid levels with CVD mortality were conducted, as detailed in Supplementary Tables S4–S8.

**Table 3:** Stratified analyses of the associations between serum lycopene concentrations and CVD mortality among middle-aged and elderly adults with MetS in NHANES III and NHANES 2001–2006.

	Serum lycopene (μmol/L)					
	Quartile 1	Quartile 2	Quartile 3	Quartile 4	*P* trend	*P* interaction
Characteristics	<0.242	0.242–0.373	0.373–0.522	>0.522		
Age, years						0.578
≤60 (*n* = 3,165)	1	0.86 (0.52, 1.42)	0.76 (0.45, 1.26)	0.81 (0.49, 1.34)	0.360	
>60 (*n* = 3,436)	1	0.83 (0.69, 0.99)	0.84 (0.65, 1.10)	0.63 (0.47, 0.84)	0.005	
Sex						0.595
Male (*n* = 2,954)	1	0.72 (0.54, 0.96)	0.80 (0.57, 1.12)	0.78 (0.54, 1.12)	0.293	
Female (*n* = 3,647)	1	0.88 (0.71, 1.11)	0.79 (0.59, 1.04)	0.58 (0.41, 0.83)	0.003	
Smoking status						0.065
Current (*n* = 1,263)	1	0.83 (0.50, 1.37)	0.42 (0.24, 0.74)	0.73 (0.34, 1.54)	0.188	
Never/past (*n* = 5,338)	1	0.81 (0.67, 0.98)	0.91 (0.74, 1.12)	0.64 (0.50, 0.81)	0.002	
Physical activity						0.083
Inactive (*n* = 3,608)	1	0.90 (0.71, 1.14)	0.65 (0.52, 0.80)	0.59 (0.42, 0.84)	0.001	
Active/ insufficient (*n* = 2,993)	1	0.70 (0.53, 0.92)	0.91 (0.66, 1.24)	0.72 (0.51, 1.02)	0.209	
eGFR, mL/min per 1.73 m^2^						0.428
≤60 (*n* = 2,017)	1	0.80 (0.62, 1.02)	0.83 (0.62, 1.11)	0.79 (0.57, 1.12)	0.235	
>60 (*n* = 4,584)	1	0.80 (0.63, 1.03)	0.76 (0.58, 0.98)	0.60 (0.44, 0.82)	0.002	
MetS components						0.924
3 (*n* = 3,288)	1	0.69 (0.49, 0.96)	0.82 (0.61, 1.11)	0.66 (0.45, 0.98)	0.108	
4 (*n* = 2,349)	1	1.03 (0.74, 1.44)	0.83 (0.60, 1.15)	0.77 (0.51, 1.17)	0.124	
5 (*n* = 964)	1	0.67 (0.48, 0.94)	0.70 (0.45, 1.07)	0.55 (0.33, 0.92)	0.034	

Data are presented as HR (95% CI). Adjusted for age (continuous), sex (male or female), race (non-Hispanic white or other), education level (less than high school, high school or equivalent, or college or above), smoking status (never smoker, former smoker, or current smoker), alcohol consumption status (nondrinker, low-to-moderate drinker, or heavy drinker), family poverty income ratio (<1.0, 1.0–3.0, or > 3.0), physical activity (inactive group, insufficiently active group, or active group), supplement use (yes or no), total energy intakes (in quartiles), eGFR (continuous), BMI(continuous), SBP (continuous), serum non-HDL-C (continuous), fasting glucose (continuous), Mets components (3–5), insulin use (yes or no), diabetic pills use (yes or no), antihypertensive medication use (yes or no), antihyperlipidemic drug use (yes or no).

In sensitivity analyses, the negative association between serum lycopene levels and CVD mortality was not materially changed when subjects who died within the initial 24 months of follow-up were excluded (Supplementary Table S3). After additional adjustments for individual dietary elements, including intakes of total fat, total protein, cholesterol, folate, fiber, and vitamins A, E, C, and B12 levels (all in quartiles), the results remained largely consistent. Consistent results persisted after additional adjustment for quartiles of serum nutrient biomarker concentrations, including vitamins A, C, D, and E (Supplementary Table S9).

## Discussion

In this prospective study, we examined the relationship between serum concentrations of five major carotenoids and the risk of CVD mortality among 6,601 metabolic syndrome-afflicted middle-aged and older subjects over a median follow-up duration of 16.8 years. Our analyses revealed an inverse dose–response relationship between serum lycopene concentrations and the risk of CVD mortality. This inverse relationship was independent of traditional risk elements, including dietary intake, lifestyle behaviors, MetS components, and kidney function as estimated by the glomerular filtration rate. In contrast, high circulating levels of *α*-carotene, *β*-cryptoxanthin, β-carotene, and combined lutein/zeaxanthin were not notably connected to CVD mortality risk among metabolic syndrome-afflicted 40-year-old and older adults in the present study. Multiple sensitivity analyses and stratified analyses confirmed the robustness of our findings.

Oxidative stress (OS) is characterized by excess ROS, leading to redox signaling disruptions and molecular damage (29). Increased OS in adipose tissue has been identified as a significant early contributor to MetS (30, 31). Research involving obese murine models has demonstrated that the administration of oxidase inhibitors leads to increased adiponectin expression and decreased TNF-*α* expression, thereby mitigating complications such as diabetes, hyperlipidemia, and hepatic steatosis (32). Carotenoids, acting as potent scavengers of ROS, possess inherent antioxidant properties, which constitute their primary beneficial effects (33). Lycopene is primarily stored in adipose tissue and exhibits superior efficacy in neutralizing singlet oxygen and free radicals compared to other carotenoids, including *β*-cryptoxanthin, β-carotene, lutein, and zeaxanthin (30, 34, 35). Specifically, lycopene demonstrates tenfold greater potency than *α*-tocopherol and twice the potency of β-carotene in counteracting OS (34). Recent preclinical studies indicate that lycopene regulates key signaling pathways, including AGE/RAGE, JNK/MAPK, PI3K/Akt, and SIRT1/FoxO1/PPARγ, resulting in a reduction in the production of pro-inflammatory markers (30, 36).

Observational studies suggested an inverse association between elevated plasma lycopene concentrations and the risk of cardiovascular disease, as well as a favorable impact on dysregulated metabolic phenotypes (14, 18, 37–40). According to Müller et al., lycopene supplementation is particularly advantageous for individuals with antioxidant deficiencies, such as the elderly, as well as those experiencing heightened oxidative stress, including smokers, diabetics, and post-myocardial infarction patients (41). Furthermore, a cross-sectional study of middle-aged and elderly men found an inverse association between lycopene levels and the prevalence of MetS, waist circumference, and triglycerides (14). Lycopene has been shown to reduce LDL oxidation, although this effect does not necessarily correlate with decreased LDL cholesterol levels (35, 36, 42). Among carotenoids, only lycopene has been found to modulate the expression of adhesion molecules in cultured human aortic endothelial cells (43). Furthermore, lycopene may decrease Rho-associated kinase expression and regulate the NO/cGMP signaling pathway, thereby mitigating atherosclerosis (44). In our study, the average lycopene concentration was 0.4 μmol/L, which is within the median range of previously reported plasma lycopene concentrations (13, 18, 35, 45–48). Research indicates that a lycopene concentration of 0.4 μmol/L is associated with the lowest all-cause mortality rate, potentially elucidating one reason for the robust correlation observed between lycopene levels in our results and CVD mortality (48). Moreover, our research demonstrates that low total carotenoid levels, along with low *α*-carotene and lutein/zeaxanthin concentrations, are significantly associated with decreased CVD mortality, consistent with previous reports (13, 48). These results extend earlier findings regarding the cardiovascular effects of carotenoids in populations with MetS. Further prospective research involving larger sample sizes is essential to explore the identified associations more comprehensively.

Additionally, subgroup analyses of serum carotenoids revealed significant interactions between lutein/zeaxanthin and *β*-cryptoxanthin with the eGFR stage. Specifically, these carotenoids exhibited protective effects in individuals with an eGFR greater than 60 mL/min/1.73m^2^, while promoting CVD mortality in those with an eGFR less than 60 mL/min/1.73m^2^. This phenomenon may be attributed to the oxidative-reductive status of patients, as substantial evidence indicates that OS is heightened in chronic kidney disease patients (33, 49). Under conditions of elevated intracellular OS, increased oxygen tension, and low levels of endogenous antioxidants, carotenoids can act as pro-oxidants (33, 50). As previously mentioned, among the diabetic population, elevated circulating *β*-carotene levels have shown a significant positive association with increased cardiovascular disease risk (13). Moreover, high-dose β-carotene supplementation has been linked to a higher incidence of lung cancer among male smokers (22). The interaction of carotenoids with varying oxidative-reductive states, along with their effects on specific organelles or tissues, suggests that the overall impact of carotenoids may differ markedly (51).

Our study has several notable strengths. Firstly, utilizing a large, multiethnic cohort with an extended follow-up period, we identified an inverse dose–response relationship between serum lycopene levels and CVD mortality among middle-aged and elderly men with MetS. Secondly, we adjusted for multiple potential confounders, including lifestyle factors, medication use, and components of MetS, thereby ensuring robust control of confounding effects on our findings. Importantly, our results remained consistent after accounting for dietary factors and other serum nutrient levels, suggesting that the observed association is not merely a reflection of overall nutritional status or supplement use. Furthermore, our sensitivity analyses provide on the results of serum lycopene as an independent biomarker for long-term CVD mortality risk in this population. These findings highlight the need for optimizing dietary structures and addressing nutritional insufficiencies, particularly in populations with decreased lycopene levels.

However, several limitations should be acknowledged. First, the observational study design limits our ability to establish causal or temporal relationships between serum lycopene levels and CVD mortality. Second, similar to previous studies (18), while serum carotenoid concentrations may reflect average daily intake, our analysis relies on a single baseline measurement, which does not capture potential fluctuations in these levels over the follow-up period. Finally, despite adjusting for multiple potential confounders in our analyses, we cannot entirely exclude the possibility of residual or unmeasured confounding.

## Conclusion

In this nationally representative sample of US middle-aged and elderly men with MetS, higher serum lycopene concentrations were associated with lower cardiovascular mortality risk. This relationship was not observed for other carotenoids. While these findings suggest a potential role for lycopene in cardiovascular risk assessment, further research is needed to confirm and elucidate this association.

## Data Availability

Publicly available datasets were analyzed in this study. This data can be found at: NHANES (https://wwwn.cdc.gov/nchs/nhanes/default.aspx).

## Footnotes

^1^ https://wwwn.cdc.gov/Nchs/Nhanes/2003-2004/L45VIT_C.htm

^2^ https://www.cdc.gov/nchs/nhanes/
