# Supplementary material for: Impact of serum carotenoids on cardiovascular mortality risk in middle-aged and elderly adults with metabolic syndrome
Source: Front Nutr. 2024 Nov 13;11:1465972. doi: 10.3389/fnut.2024.1465972 (PMC11598320; doi:10.3389/fnut.2024.1465972)

SUPPLEMENTAL MATERIAL

Impact of Serum Carotenoids on Cardiovascular Mortality Risk in Middle-Aged and Elderly Adults with Metabolic Syndrome

Table S1 Distributions and concentrations of serum carotenoids among middle-aged and elderly adults with MetS in NHANES III and NHANES 2001–2006

| Serum carotenoids | Mean | 5^th^ | 25^th^ | 50^th^ | 75^th^ | 95^th^ |
| --- | --- | --- | --- | --- | --- | --- |
| α-Carotene, μmol/L | 0.079 | 0.015 | 0.032 | 0.058 | 0.097 | 0.205 |
| β-Carotene, μmol/L | 0.350 | 0.075 | 0.149 | 0.252 | 0.421 | 0.932 |
| β-Cryptoxanthin, μmol/L | 0.158 | 0.044 | 0.082 | 0.127 | 0.199 | 0.373 |
| Lycopene, μmol/L | 0.400 | 0.112 | 0.242 | 0.373 | 0.522 | 0.784 |
| Lutein/zeaxanthin, μmol/L | 0.337 | 0.123 | 0.204 | 0.290 | 0.418 | 0.687 |
| Total carotenoids, μmol/L | 1.324 | 0.527 | 0.868 | 1.189 | 1.616 | 2.494 |

Table S2 Hazard ratios (95% CIs) of CVD according to quartiles of serum total carotenoids concentrations among middle-aged and elderly adults with MetS in NHANES III and NHANES 2001–2006

|  | Serum total carotenoids (μmol/L) | | | |  |
| --- | --- | --- | --- | --- | --- |
|  | Quartile 1 | Quartile 2 | Quartile 3 | Quartile 4 | *P* trend |
| Range | <0.868 | 0.868- 1.189 | 1.189-1.616 | >1.616 |  |
| No.deaths/total | 272/1531 | 267/1568 | 309/1676 | 389/1826 |  |
| Crude | 1 | 0.65 (0.52, 0.81) | 0.75 (0.59, 0.96) | 0.88 (0.72, 1.09) | 0.558 |
| Model 1 | 1 | 0.76 (0.59, 0.98) | 0.82 (0.64, 1.05) | 0.83 (0.66, 1.04) | 0.160 |
| Model 2 | 1 | 0.75 (0.59, 0.96) | 0.83 (0.65, 1.06) | 0.83 (0.66, 1.04) | 0.165 |

Model 1 was adjusted for age (continuous), sex (male or female), race (non-Hispanic white or other), education level (less than high school, high school or equivalent, or college or above), smoking status (never smoker, former smoker, or current smoker), alcohol consumption status (nondrinker, low-to-moderate drinker, or heavy drinker), family poverty income ratio (<1.0, 1.0–3.0, or >3.0), physical activity (inactive group, insufficiently active group, or active group), supplement use (yes or no), total energy intakes (in quartiles), eGFR (continuous), BMI(continuous).

Model 2 was adjusted for as model 1 plus SBP (continuous), serum non-HDL-C (continuous), fasting glucose (continuous), Mets components (3-5), insulin use (yes or no), diabetic pills use (yes or no), antihypertensive medication use (yes or no), antihyperlipidemic drug use (yes or no).

Table S3 Hazard ratios (95% CIs) of CVD according to quartiles of serum carotenoids concentrations among middle-aged and elderly adults with MetS after excluding participants who died within two years of follow-up in NHANES III and NHANES 2001–2006 (n = 6460)

|  | Serum carotenoids (μmol/L) | | | |  |
| --- | --- | --- | --- | --- | --- |
|  | Quartile 1 | Quartile 2 | Quartile 3 | Quartile 4 | *P* trend |
| Lycopene |  |  |  |  |  |
| Range | <0.242 | 0.242-0.373 | 0.373-0.522 | >0.522 |  |
| No.deaths/total | 446/2003 | 320/1789 | 242/1416 | 174/1252 |  |
| Crude | 1 | 0.64 (0.53, 0.77) | 0.50 (0.42, 0.61) | 0.40 (0.32, 0.50) | <0.001 |
| Model 1 | 1 | 0.86 (0.72, 1.02) | 0.86 (0.73, 1.02) | 0.77 (0.62, 0.97) | 0.028 |
| Model 2 | 1 | 0.86 (0.72, 1.02) | 0.89 (0.76, 1.05) | 0.77 (0.61, 0.98) | 0.052 |
| α-Carotene |  |  |  |  |  |
| Range | <0.032 | 0.032- 0.058 | 0.058-0.097 | >0.097 |  |
| No. deaths/total | 248/1514 | 261/1613 | 334/1698 | 339/1635 |  |
| Crude | 1 | 0.92 (0.73, 1.15) | 1.05 (0.85, 1.30) | 1.14 (0.92, 1.40) | 0.146 |
| Model 1 | 1 | 0.79 (0.63, 0.99) | 0.88 (0.70, 1.10) | 0.83 (0.66, 1.05) | 0.255 |
| Model 2 | 1 | 0.76 (0.61, 0.95) | 0.84 (0.67, 1.06) | 0.83 (0.65, 1.06) | 0.262 |
| β-Carotene |  |  |  |  |  |
| Range | <0.149 | 0.149-0.252 | 0.252-0.421 | >0.421 |  |
| No. deaths/total | 220/1513 | 249/1528 | 315/1643 | 398/1776 |  |
| Crude | 1 | 1.13 (0.83, 1.54) | 1.31 (1.01, 1.70) | 1.56 (1.24, 1.98) | <0.001 |
| Model 1 | 1 | 0.96 (0.69, 1.34) | 1.00 (0.76, 1.31) | 0.88 (0.67, 1.16) | 0.429 |
| Model 2 | 1 | 0.94 (0.67, 1.32) | 1.04 (0.80, 1.35) | 0.93 (0.72, 1.19) | 0.736 |
| β-Cryptoxanthin |  |  |  |  |  |
| Range | <0.081 | 0.081-0.127 | 0.127-0.199 | >0.199 |  |
| No. deaths/total | 234/1298 | 322/1656 | 264/1498 | 362/2008 |  |
| Crude | 1 | 0.98 (0.79, 1.23) | 0.85 (0.67, 1.07) | 0.97 (0.79, 1.19) | 0.454 |
| Model 1 | 1 | 1.03 (0.82, 1.30) | 0.89 (0.69, 1.14) | 0.85 (0.68, 1.06) | 0.074 |
| Model 2 | 1 | 1.00 (0.79, 1.28) | 0.87 (0.68, 1.11) | 0.83 (0.67, 1.04) | 0.044 |
| Lutein/zeaxanthin |  |  |  |  |  |
| Range | <0.204 | 0.204-0.290 | 0.290-0.418 | >0.418 |  |
| No. deaths/total | 181/1246 | 248/1538 | 296/1718 | 457/1958 |  |
| Crude | 1 | 1.04 (0.78, 1.38) | 0.97 (0.76, 1.25) | 1.27 (0.98, 1.64) | 0.126 |
| Model 1 | 1 | 0.83 (0.65, 1.07) | 0.78 (0.60, 1.01) | 0.88 (0.70, 1.11) | 0.287 |
| Model 2 | 1 | 0.80 (0.63, 1.01) | 0.76 (0.58, 1.00) | 0.82 (0.64, 1.06) | 0.152 |

Model 1 was adjusted for age (continuous), sex (male or female), race (non-Hispanic white or other), education level (less than high school, high school or equivalent, or college or above), smoking status (never smoker, former smoker, or current smoker), alcohol consumption status (nondrinker, low-to-moderate drinker, or heavy drinker), family poverty income ratio (<1.0, 1.0–3.0, or >3.0), physical activity (inactive group, insufficiently active group, or active group), supplement use (yes or no), total energy intakes (in quartiles), eGFR (continuous), BMI (continuous).

Model 2 was adjusted for as model 1 plus SBP (continuous), serum non-HDL-C (continuous), fasting glucose (continuous), Mets components (3-5), insulin use (yes or no), diabetic pills use (yes or no), antihypertensive medication use (yes or no), antihyperlipidemic drug use (yes or no).

Table S4 Stratified analyses of the associations between serum α-carotene concentrations and CVD mortality among middle-aged and elderly adults with MetS in NHANES III and NHANES 2001–2006

|  | Serum α-carotene (μmol/L) | | | | |  |
| --- | --- | --- | --- | --- | --- | --- |
|  | Quartile 1 | Quartile 2 | Quartile 3 | Quartile 4 |  |  |
| Characteristics | <0.032 | 0.032- 0.058 | 0.058-0.097 | >0.097 | *P* _trend_ | *P* _interaction_ |
| Age, years |  |  |  |  |  | 0.300 |
| ≤60 (n = 3,165) | 1 | 0.75 (0.50, 1.12) | 0.89 (0.53, 1.47) | 0.76 (0.47, 1.23) | 0.451 |  |
| >60 (n = 3,436) | 1 | 0.84 (0.60, 1.18) | 0.97 (0.71, 1.32) | 0.92 (0.69, 1.23) | 0.786 |  |
| Sex |  |  |  |  |  | 0.189 |
| Male (n = 2,954) | 1 | 1.06 (0.75, 1.50) | 1.14 (0.84, 1.54) | 1.10 (0.80, 1.50) | 0.503 |  |
| Female (n = 3,647) | 1 | 0.57 (0.41, 0.79) | 0.73 (0.54, 1.00) | 0.69 (0.51, 0.92) | 0.085 |  |
| Smoking status |  |  |  |  |  | 0.634 |
| Current (n = 1,263) | 1 | 0.75 (0.48, 1.17) | 0.90 (0.47, 1.72) | 0.63 (0.28, 1.39) | 0.367 |  |
| Never/past (n = 5,338) | 1 | 0.84 (0.64, 1.11) | 0.97 (0.79, 1.19) | 0.91 (0.72, 1.17) | 0.710 |  |
| Physical activity |  |  |  |  |  | 0.712 |
| Inactive (n = 3,608) | 1 | 0.80 (0.61, 1.05) | 0.83 (0.63, 1.09) | 0.84 (0.63, 1.11) | 0.270 |  |
| Active/ insufficient (n = 2,993) | 1 | 0.74 (0.48, 1.15) | 1.05 (0.72, 1.53) | 0.90 (0.60, 1.33) | 0.966 |  |
| eGFR, mL/min per 1.73 m2 |  |  |  |  |  | 0.330 |
| ≤60 (n = 2,017) | 1 | 0.70 (0.45, 1.07) | 0.93 (0.65, 1.32) | 0.89 (0.61, 1.29) | 0.890 |  |
| >60 (n = 4,584) | 1 | 0.93 (0.69, 1.24) | 0.96 (0.71, 1.29) | 0.88 (0.62, 1.27) | 0.596 |  |
| MetS components |  |  |  |  |  | 0.230 |
| 3 (n = 3,288) | 1 | 0.75 (0.50, 1.12) | 0.85 (0.57, 1.26) | 0.71 (0.47, 1.05) | 0.155 |  |
| 4 (n = 2,349) | 1 | 0.73 (0.48, 1.09) | 0.83 (0.62, 1.11) | 0.98 (0.65, 1.47) | 0.921 |  |
| 5 (n = 964) | 1 | 1.18 (0.78, 1.78) | 1.39 (0.79, 2.44) | 1.25 (0.79, 1.99) | 0.320 |  |

Data are presented as HR (95% CI). Adjusted for age (continuous), sex (male or female), race (non-Hispanic white or other), education level (less than high school, high school or equivalent, or college or above), smoking status (never smoker, former smoker, or current smoker), alcohol consumption status (nondrinker, low-to-moderate drinker, or heavy drinker), family poverty income ratio (<1.0, 1.0–3.0, or >3.0), physical activity (inactive group, insufficiently active group, or active group), supplement use (yes or no), total energy intakes (in quartiles), eGFR (continuous), BMI(continuous), SBP (continuous), serum non-HDL-C (continuous), fasting glucose (continuous), Mets components (3-5), insulin use (yes or no), diabetic pills use (yes or no), antihypertensive medication use (yes or no), antihyperlipidemic drug use (yes or no).

Table S5 Stratified analyses of the associations between serum β-carotene concentrations and CVD mortality among middle-aged and elderly adults with MetS in NHANES III and NHANES 2001–2006

|  | Serum β-Carotene (μmol/L) | | | | |  |
| --- | --- | --- | --- | --- | --- | --- |
|  | Quartile 1 | Quartile 2 | Quartile 3 | Quartile 4 |  |  |
| Characteristics | <0.149 | 0.149-0.252 | 0.252-0.421 | >0.421 | *P* _trend_ | *P* _interaction_ |
| Age, years |  |  |  |  |  | 0.134 |
| ≤60 (n = 3,165) | 1 | 0.83 (0.48, 1.41) | 0.95 (0.54, 1.66) | 0.85 (0.51, 1.41) | 0.682 |  |
| >60 (n = 3,436) | 1 | 1.12 (0.80, 1.56) | 1.34 (1.02, 1.76) | 1.24 (0.95, 1.63) | 0.042 |  |
| Sex |  |  |  |  |  | 0.766 |
| Male (n = 2,954) | 1 | 0.90 (0.62, 1.32) | 1.24 (0.90, 1.73) | 1.22 (0.88, 1.69) | 0.092 |  |
| Female (n = 3,647) | 1 | 0.96 (0.64, 1.44) | 1.01 (0.73, 1.39) | 0.92 (0.65, 1.30) | 0.704 |  |
| Smoking status |  |  |  |  |  | 0.303 |
| Current (n = 1,263) | 1 | 0.84 (0.46, 1.52) | 1.07 (0.53, 2.15) | 0.72 (0.35, 1.46) | 0.524 |  |
| Never/past (n = 5,338) | 1 | 1.00 (0.68, 1.48) | 1.15 (0.88, 1.50) | 1.13 (0.83, 1.52) | 0.270 |  |
| Physical activity |  |  |  |  |  | 0.553 |
| Inactive (n = 3,608) | 1 | 0.98 (0.68, 1.40) | 1.21 (0.89, 1.64) | 1.03 (0.74, 1.43) | 0.542 |  |
| Active/ insufficient (n = 2,993) | 1 | 0.81 (0.48, 1.38) | 0.91 (0.61, 1.35) | 0.97 (0.65, 1.45) | 0.988 |  |
| eGFR, mL/min per 1.73 m2 |  |  |  |  |  | 0.020 |
| ≤60 (n = 2,017) | 1 | 1.01 (0.69, 1.48) | 1.30 (0.91, 1.84) | 1.37 (0.95, 1.98) | 0.039 |  |
| >60 (n = 4,584) | 1 | 0.94 (0.62, 1.41) | 1.10 (0.74, 1.62) | 0.87 (0.59, 1.30) | 0.693 |  |
| MetS components |  |  |  |  |  | 0.858 |
| 3 (n = 3,288) | 1 | 1.14 (0.77, 1.69) | 1.17 (0.76, 1.80) | 1.14 (0.74, 1.75) | 0.574 |  |
| 4 (n = 2,349) | 1 | 0.73 (0.45, 1.17) | 1.24 (0.85, 1.83) | 1.06 (0.75, 1.50) | 0.212 |  |
| 5 (n = 964) | 1 | 0.96 (0.56, 1.65) | 0.80 (0.50, 1.30) | 0.85 (0.47, 1.53) | 0.449 |  |

Data are presented as HR (95% CI). Adjusted for age (continuous), sex (male or female), race (non-Hispanic white or other), education level (less than high school, high school or equivalent, or college or above), smoking status (never smoker, former smoker, or current smoker), alcohol consumption status (nondrinker, low-to-moderate drinker, or heavy drinker), family poverty income ratio (<1.0, 1.0–3.0, or >3.0), physical activity (inactive group, insufficiently active group, or active group), supplement use (yes or no), total energy intakes (in quartiles), eGFR (continuous), BMI(continuous), SBP (continuous), serum non-HDL-C (continuous), fasting glucose (continuous), Mets components (3-5), insulin use (yes or no), diabetic pills use (yes or no), antihypertensive medication use (yes or no), antihyperlipidemic drug use (yes or no).

Table S6 Stratified analyses of the associations between serum β-cryptoxanthin concentrations and CVD mortality among middle-aged and elderly adults with MetS in NHANES III and NHANES 2001–2006

|  | Serum β-cryptoxanthin (μmol/L) | | | | |  |
| --- | --- | --- | --- | --- | --- | --- |
|  | Quartile 1 | Quartile 2 | Quartile 3 | Quartile 4 |  |  |
| Characteristics | <0.081 | 0.081-0.127 | 0.127-0.199 | >0.199 | *P* _trend_ | *P* _interaction_ |
| Age, years |  |  |  |  |  | 0.034 |
| ≤60 (n = 3,165) | 1 | 1.05 (0.73, 1.52) | 0.72 (0.45, 1.18) | 0.56 (0.32, 1.00) | 0.034 |  |
| >60 (n = 3,436) | 1 | 1.09 (0.82, 1.45) | 1.03 (0.79, 1.34) | 1.09 (0.84, 1.42) | 0.632 |  |
| Sex |  |  |  |  |  | 0.989 |
| Male (n = 2,954) | 1 | 1.08 (0.80, 1.48) | 0.96 (0.68, 1.36) | 1.08 (0.80, 1.45) | 0.830 |  |
| Female (n = 3,647) | 1 | 0.97 (0.73, 1.29) | 0.89 (0.65, 1.20) | 0.89 (0.64, 1.23) | 0.413 |  |
| Smoking status |  |  |  |  |  | 0.979 |
| Current (n = 1,263) | 1 | 1.24 (0.74, 2.08) | 0.91 (0.45, 1.83) | 0.92 (0.52, 1.64) | 0.549 |  |
| Never/past (n = 5,338) | 1 | 1.02 (0.75, 1.38) | 0.90 (0.67, 1.21) | 0.90 (0.71, 1.14) | 0.238 |  |
| Physical activity |  |  |  |  |  | 0.793 |
| Inactive (n = 3,608) | 1 | 1.00 (0.75, 1.33) | 0.84 (0.61, 1.14) | 0.92 (0.66, 1.28) | 0.425 |  |
| Active/ insufficient (n = 2,993) | 1 | 1.16 (0.82, 1.64) | 1.05 (0.74, 1.49) | 1.04 (0.77, 1.41) | 0.971 |  |
| eGFR, mL/min per 1.73 m2 |  |  |  |  |  | 0.018 |
| ≤60 (n = 2,017) | 1 | 1.35 (0.98, 1.85) | 1.21 (0.91, 1.62) | 1.35 (1.01, 1.81) | 0.093 |  |
| >60 (n = 4,584) | 1 | 0.94 (0.68, 1.29) | 0.75 (0.50, 1.13) | 0.69 (0.50, 0.94) | 0.016 |  |
| MetS components |  |  |  |  |  | 0.841 |
| 3 (n = 3,288) | 1 | 0.94 (0.62, 1.43) | 0.78 (0.52, 1.18) | 0.83 (0.58, 1.19) | 0.192 |  |
| 4 (n = 2,349) | 1 | 1.16 (0.76, 1.78) | 0.96 (0.65, 1.42) | 1.09 (0.72, 1.64) | 0.915 |  |
| 5 (n = 964) | 1 | 1.09 (0.64, 1.84) | 1.04 (0.61, 1.75) | 0.82 (0.44, 1.53) | 0.492 |  |

Data are presented as HR (95% CI). Adjusted for age (continuous), sex (male or female), race (non-Hispanic white or other), education level (less than high school, high school or equivalent, or college or above), smoking status (never smoker, former smoker, or current smoker), alcohol consumption status (nondrinker, low-to-moderate drinker, or heavy drinker), family poverty income ratio (<1.0, 1.0–3.0, or >3.0), physical activity (inactive group, insufficiently active group, or active group), supplement use (yes or no), total energy intakes (in quartiles), eGFR (continuous), BMI(continuous), SBP (continuous), serum non-HDL-C (continuous), fasting glucose (continuous), Mets components (3-5), insulin use (yes or no), diabetic pills use (yes or no), antihypertensive medication use (yes or no), antihyperlipidemic drug use (yes or no).

Table S7 Stratified analyses of the associations between serum lutein/zeaxanthin concentrations and CVD mortality among middle-aged and elderly adults with MetS in NHANES III and NHANES 2001–2006

|  | Serum lutein/zeaxanthin (μmol/L) | | | | |  |
| --- | --- | --- | --- | --- | --- | --- |
|  | Quartile 1 | Quartile 2 | Quartile 3 | Quartile 4 |  |  |
| Characteristics | <0.204 | 0.204-0.290 | 0.290-0.418 | >0.418 | *P* _trend_ | *P* _interaction_ |
| Age, years |  |  |  |  |  | 0.178 |
| ≤60 (n = 3,165) | 1 | 0.75 (0.44, 1.28) | 0.81 (0.46, 1.41) | 0.64 (0.36, 1.12) | 0.195 |  |
| >60 (n = 3,436) | 1 | 0.90 (0.70, 1.15) | 0.81 (0.62, 1.06) | 1.05 (0.81, 1.36) | 0.917 |  |
| Sex |  |  |  |  |  | 0.472 |
| Male (n = 2,954) | 1 | 0.90 (0.63, 1.29) | 0.84 (0.57, 1.25) | 0.90 (0.57, 1.41) | 0.605 |  |
| Female (n = 3,647) | 1 | 0.81 (0.58, 1.15) | 0.83 (0.59, 1.16) | 1.00 (0.73, 1.38) | 0.958 |  |
| Smoking status |  |  |  |  |  | 0.857 |
| Current (n = 1,263) | 1 | 1.19 (0.74, 1.92) | 0.92 (0.47, 1.79) | 1.33 (0.64, 2.75) | 0.601 |  |
| Never/past (n = 5,338) | 1 | 0.80 (0.60, 1.07) | 0.84 (0.64, 1.10) | 0.88 (0.65, 1.18) | 0.472 |  |
| Physical activity |  |  |  |  |  | 0.069 |
| Inactive (n = 3,608) | 1 | 1.07 (0.82, 1.39) | 0.85 (0.61, 1.18) | 1.07 (0.79, 1.45) | 0.971 |  |
| Active/ insufficient (n = 2,993) | 1 | 0.55 (0.35, 0.87) | 0.74 (0.51, 1.08) | 0.71 (0.49, 1.02) | 0.208 |  |
| eGFR, mL/min per 1.73 m2 |  |  |  |  |  | 0.001 |
| ≤60 (n = 2,017) | 1 | 1.23 (0.82, 1.83) | 1.63 (1.10, 2.42) | 1.78 (1.25, 2.55) | 0.001 |  |
| >60 (n = 4,584) | 1 | 0.76 (0.56, 1.02) | 0.58 (0.43, 0.79) | 0.66 (0.44, 0.99) | 0.027 |  |
| MetS components |  |  |  |  |  | 0.303 |
| 3 (n = 3,288) | 1 | 0.82 (0.54, 1.24) | 0.70 (0.47, 1.04) | 0.99 (0.63, 1.56) | 0.797 |  |
| 4 (n = 2,349) | 1 | 0.87 (0.55, 1.37) | 0.84 (0.54, 1.31) | 0.90 (0.56, 1.44) | 0.642 |  |
| 5 (n = 964) | 1 | 0.96 (0.57, 1.61) | 1.27 (0.66, 2.43) | 1.03 (0.59, 1.80) | 0.698 |  |

Data are presented as HR (95% CI). Adjusted for age (continuous), sex (male or female), race (non-Hispanic white or other), education level (less than high school, high school or equivalent, or college or above), smoking status (never smoker, former smoker, or current smoker), alcohol consumption status (nondrinker, low-to-moderate drinker, or heavy drinker), family poverty income ratio (<1.0, 1.0–3.0, or >3.0), physical activity (inactive group, insufficiently active group, or active group), supplement use (yes or no), total energy intakes (in quartiles), eGFR (continuous), BMI(continuous), SBP (continuous), serum non-HDL-C (continuous), fasting glucose (continuous), Mets components (3-5), insulin use (yes or no), diabetic pills use (yes or no), antihypertensive medication use (yes or no), antihyperlipidemic drug use (yes or no).

Table S8 Stratified analyses of the associations between serum total carotenoids concentrations and CVD mortality among middle-aged and elderly adults with MetS in NHANES III and NHANES 2001–2006

|  | Serum total carotenoids (μmol/L) | | | | |  |
| --- | --- | --- | --- | --- | --- | --- |
|  | Quartile 1 | Quartile 2 | Quartile 3 | Quartile 4 |  |  |
| Characteristics | <0.868 | 0.868- 1.189 | 1.189-1.616 | >1.616 | *P* _trend_ | *P* _interaction_ |
| Age, years |  |  |  |  |  | 0.047 |
| ≤60 (n = 3,165) | 1 | 0.51 (0.29, 0.92) | 0.72 (0.43, 1.20) | 0.67 (0.38, 1.19) | 0.377 |  |
| >60 (n = 3,436) | 1 | 0.92 (0.71, 1.20) | 0.95 (0.77, 1.16) | 0.97 (0.74, 1.25) | 0.839 |  |
| Sex |  |  |  |  |  | 0.835 |
| Male (n = 2,954) | 1 | 0.78 (0.56, 1.07) | 0.96 (0.69, 1.34) | 1.11 (0.81, 1.52) | 0.349 |  |
| Female (n = 3,647) | 1 | 0.72 (0.55, 0.94) | 0.74 (0.53, 1.03) | 0.73 (0.54, 1.00) | 0.073 |  |
| Smoking status |  |  |  |  |  | 0.271 |
| Current (n = 1,263) | 1 | 0.61 (0.39, 0.96) | 0.52 (0.24, 1.11) | 0.81 (0.36, 1.84) | 0.555 |  |
| Never/past (n = 5,338) | 1 | 0.80 (0.60, 1.06) | 0.95 (0.76, 1.20) | 0.87 (0.69, 1.10) | 0.509 |  |
| Physical activity |  |  |  |  |  | 0.254 |
| Inactive (n = 3,608) | 1 | 0.65 (0.49, 0.88) | 0.66 (0.52, 0.83) | 0.73 (0.55, 0.98) | 0.045 |  |
| Active/ insufficient (n = 2,993) | 1 | 0.83 (0.55, 1.25) | 1.05 (0.75, 1.47) | 1.04 (0.71, 1.52) | 0.523 |  |
| eGFR, mL/min per 1.73 m2 |  |  |  |  |  | 0.009 |
| ≤60 (n = 2,017) | 1 | 1.05 (0.75, 1.46) | 1.21 (0.91, 1.62) | 1.24 (0.88, 1.74) | 0.149 |  |
| >60 (n = 4,584) | 1 | 0.61 (0.43, 0.86) | 0.66 (0.46, 0.96) | 0.71 (0.50, 1.01) | 0.113 |  |
| MetS components |  |  |  |  |  | 0.733 |
| 3 (n = 3,288) | 1 | 0.72 (0.53, 0.98) | 0.73 (0.53, 1.03) | 0.84 (0.59, 1.19) | 0.338 |  |
| 4 (n = 2,349) | 1 | 0.81 (0.51, 1.31) | 0.94 (0.62, 1.42) | 0.95 (0.64, 1.42) | 0.996 |  |
| 5 (n = 964) | 1 | 0.64 (0.40, 1.03) | 0.77 (0.48, 1.23) | 0.75 (0.43, 1.28) | 0.418 |  |

Data are presented as HR (95% CI). Adjusted for age (continuous), sex (male or female), race (non-Hispanic white or other), education level (less than high school, high school or equivalent, or college or above), smoking status (never smoker, former smoker, or current smoker), alcohol consumption status (nondrinker, low-to-moderate drinker, or heavy drinker), family poverty income ratio (<1.0, 1.0–3.0, or >3.0), physical activity (inactive group, insufficiently active group, or active group), supplement use (yes or no), total energy intakes (in quartiles), eGFR (continuous), BMI(continuous), SBP (continuous), serum non-HDL-C (continuous), fasting glucose (continuous), Mets components (3-5), insulin use (yes or no), diabetic pills use (yes or no), antihypertensive medication use (yes or no), antihyperlipidemic drug use (yes or no).

Table S9 Hazard ratios (95% CIs) of CVD according to quartiles of serum carotenoids concentrations among middle-aged and elderly adults with MetS with further adjustment of dietary factors and nutrient biomarkers in NHANES III and NHANES 2001–2006

|  | | Serum carotenoids (μmol/L) | |  | |
| --- | --- | --- | --- | --- | --- |
|  | Quartile 1 | Quartile 2 | Quartile 3 | Quartile 4 | *P* trend |
| Model + dietary factors * |  |  |  |  |  |
| α-Carotene | 1 | 0.78 (0.62, 0.99) | 0.88 (0.69, 1.11) | 0.87 (0.67, 1.14) | 0.518 |
| β-Carotene | 1 | 0.91 (0.65, 1.26) | 1.03 (0.80, 1.33) | 0.91 (0.70, 1.17) | 0.668 |
| β-Cryptoxanthin | 1 | 1.00 (0.79, 1.28) | 0.85 (0.66, 1.09) | 0.82 (0.66, 1.02) | 0.028 |
| Lycopene | 1 | 0.86 (0.72, 1.03) | 0.88 (0.75, 1.03) | 0.76 (0.60, 0.95) | 0.025 |
| Lutein/zeaxanthin | 1 | 0.77 (0.61, 0.97) | 0.73 (0.56, 0.95) | 0.79 (0.59, 1.05) | 0.118 |
| Model + nutrient biomarkers † |  |  |  |  |  |
| α-Carotene | 1 | 0.77 (0.61, 0.97) | 0.92 (0.72, 1.17) | 0.90 (0.69, 1.17) | 0.731 |
| β-Carotene | 1 | 1.06 (0.74, 1.51) | 1.14 (0.86, 1.51) | 1.11 (0.82, 1.52) | 0.406 |
| β-Cryptoxanthin | 1 | 1.06 (0.85, 1.33) | 0.85 (0.66, 1.09) | 0.90 (0.72, 1.13) | 0.154 |
| Lycopene | 1 | 0.84 (0.70, 1.00) | 0.91 (0.77, 1.08) | 0.73 (0.58, 0.93) | 0.023 |
| Lutein/zeaxanthin | 1 | 0.91 (0.69, 1.21) | 0.88 (0.65, 1.19) | 0.98 (0.71, 1.37) | 0.870 |

Model was adjusted for age (continuous), sex (male or female), race (non-Hispanic white or other), education level (less than high school, high school or equivalent, or college or above), smoking status (never smoker, former smoker, or current smoker), alcohol consumption status (nondrinker, low-to-moderate drinker, or heavy drinker), family poverty income ratio (<1.0, 1.0–3.0, or >3.0), physical activity (inactive group, insufficiently active group, or active group), supplement use (yes or no), total energy intakes (in quartiles), eGFR (continuous), BMI (continuous), SBP (continuous), serum non-HDL-C (continuous), fasting glucose (continuous), Mets components (3-5), insulin use (yes or no), diabetic pills use (yes or no), antihypertensive medication use (yes or no), antihyperlipidemic drug use (yes or no).

* Further adjusted for intakes of total protein, fat, cholesterol, fiber, folate, vitamin A, vitamin E, vitamin B12, and vitamin C (all in quartiles). There were 232 missing values for all these variables.

† Further adjusted for serum vitamin A, vitamin C, vitamin D, and vitamin E (all in quartiles). There were 1,080 missing values for all these variables.

Figure S1 Flowchart of the study participants


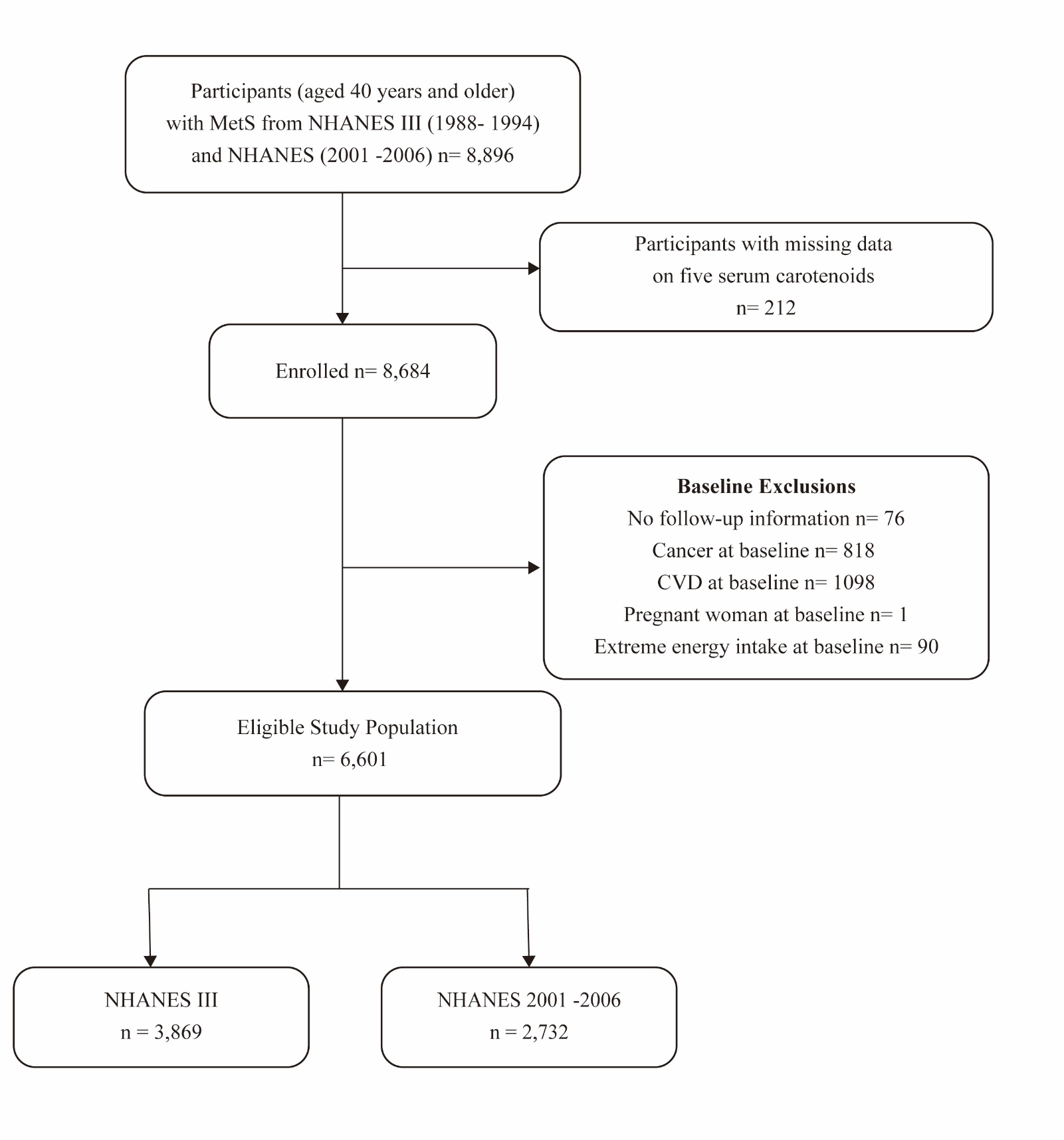


Figure S2 Kaplan-Meier Curves Depicting CVD Survival Probabilities by Quartiles of Serum Carotenoids Concentrations in Middle-Aged and Elderly Adults with MetS. (A) α-carotene; (B) β-carotene; (C) β-cryptoxanthin; (D) lycopene; (E) lutein/zeaxanthin.


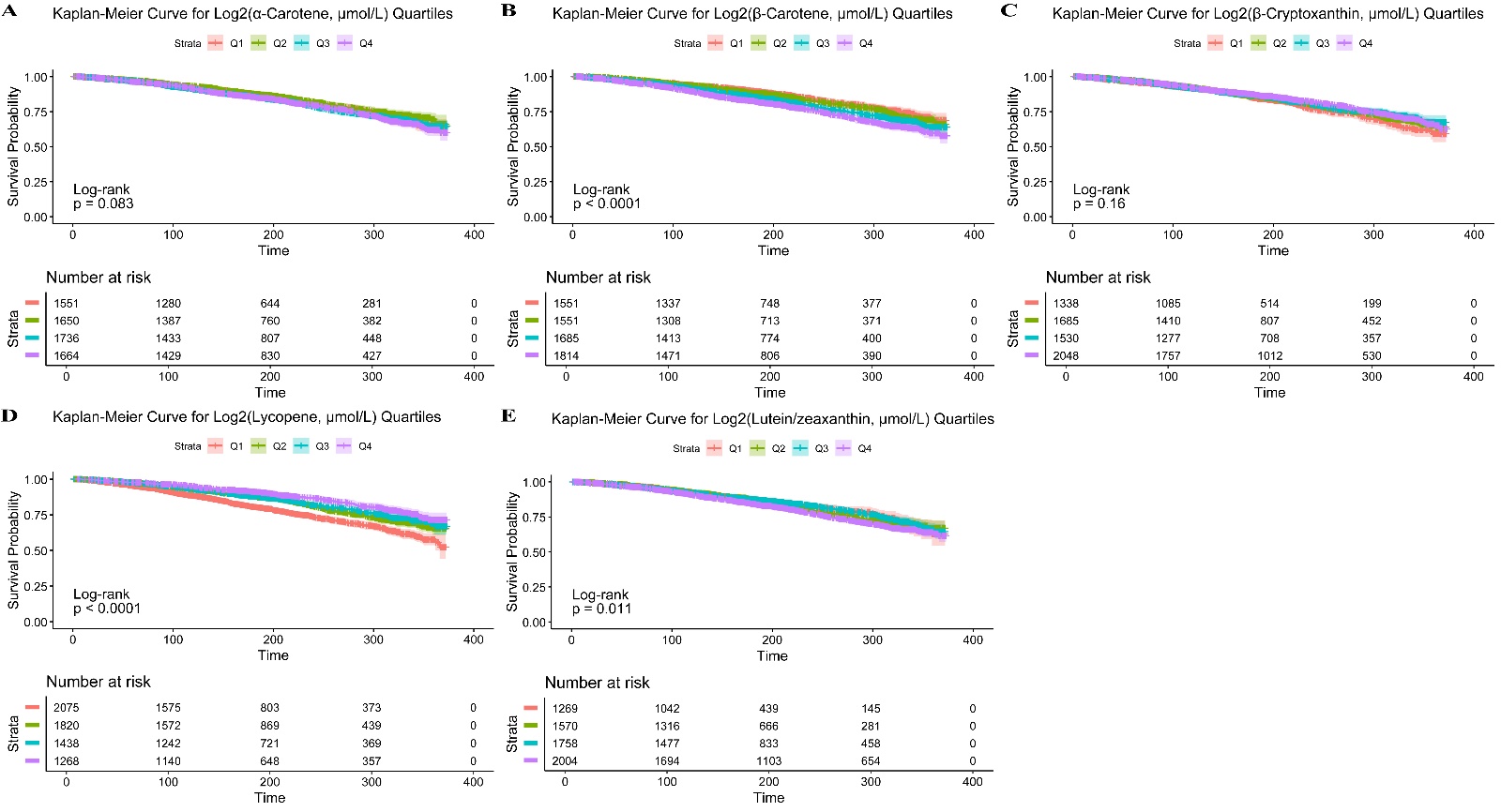

Supplement: Supplementary file 1 [file Data_Sheet_1.docx]
